# Supplementary figures and images for: De novo detection of somatic variants in high-quality long-read single-cell RNA sequencing data
Source: Genome Res. 2025 Apr;35(4):900–13. doi: 10.1101/gr.279281.124 (PMC12047253; doi:10.1101/gr.279281.124)

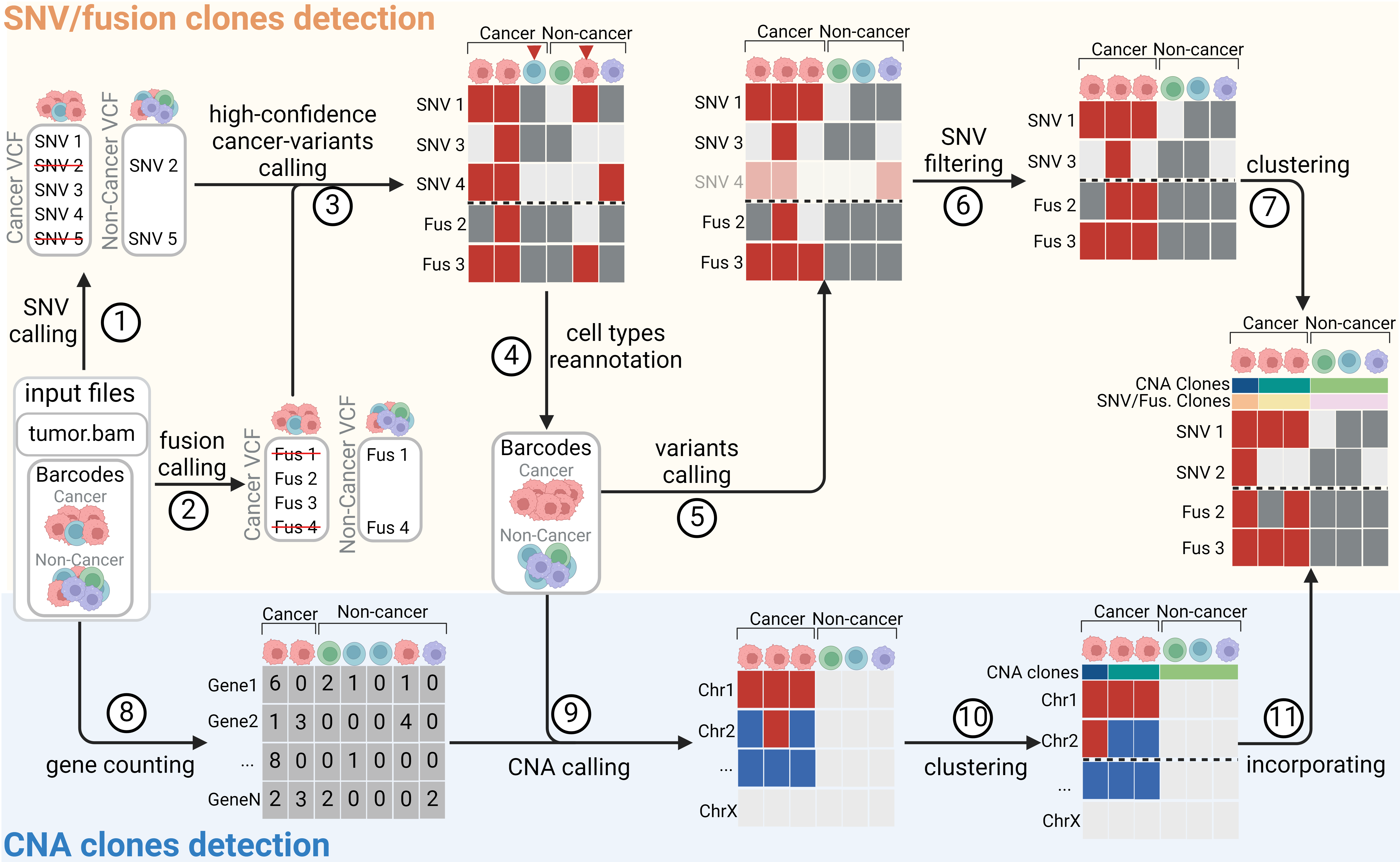

Supplement: Supplement 3 [file Supplemental_Code.zip › LongSom-paper_version/docs/Pipeline_LongSom.png]

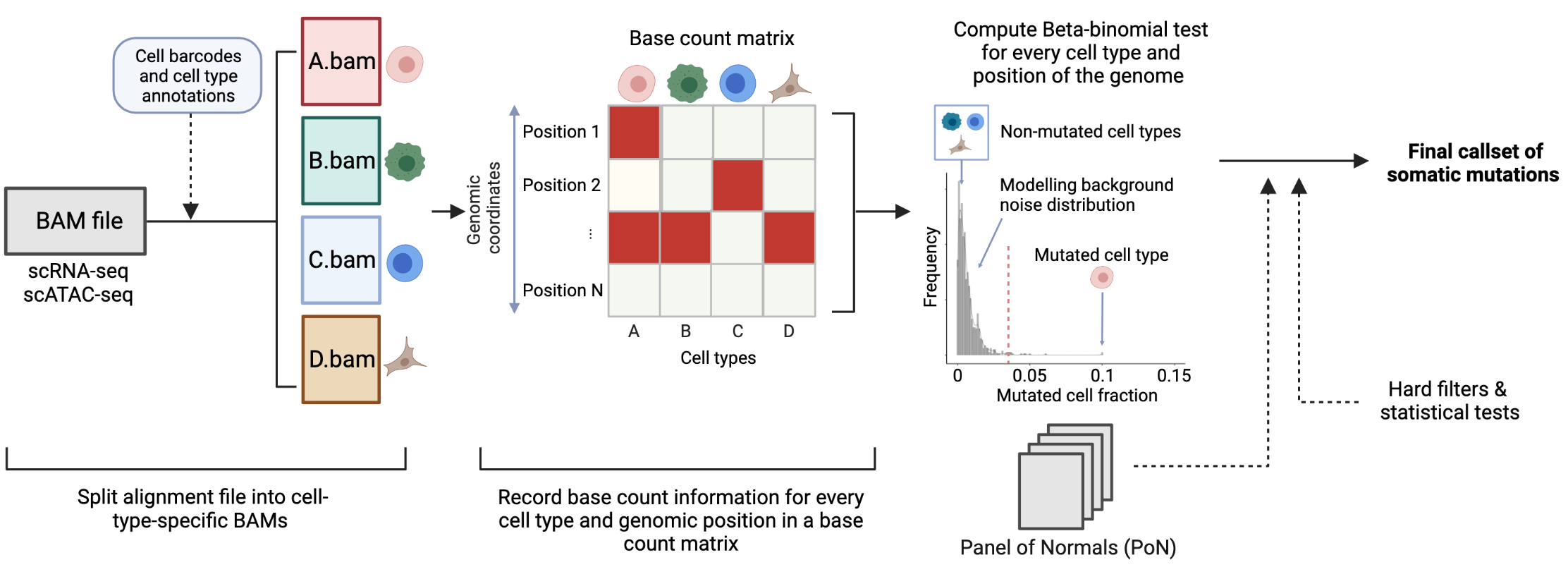

Supplement: Supplement 3 [file Supplemental_Code.zip › LongSom-paper_version/SComatic/docs/Algorithm.jpeg]

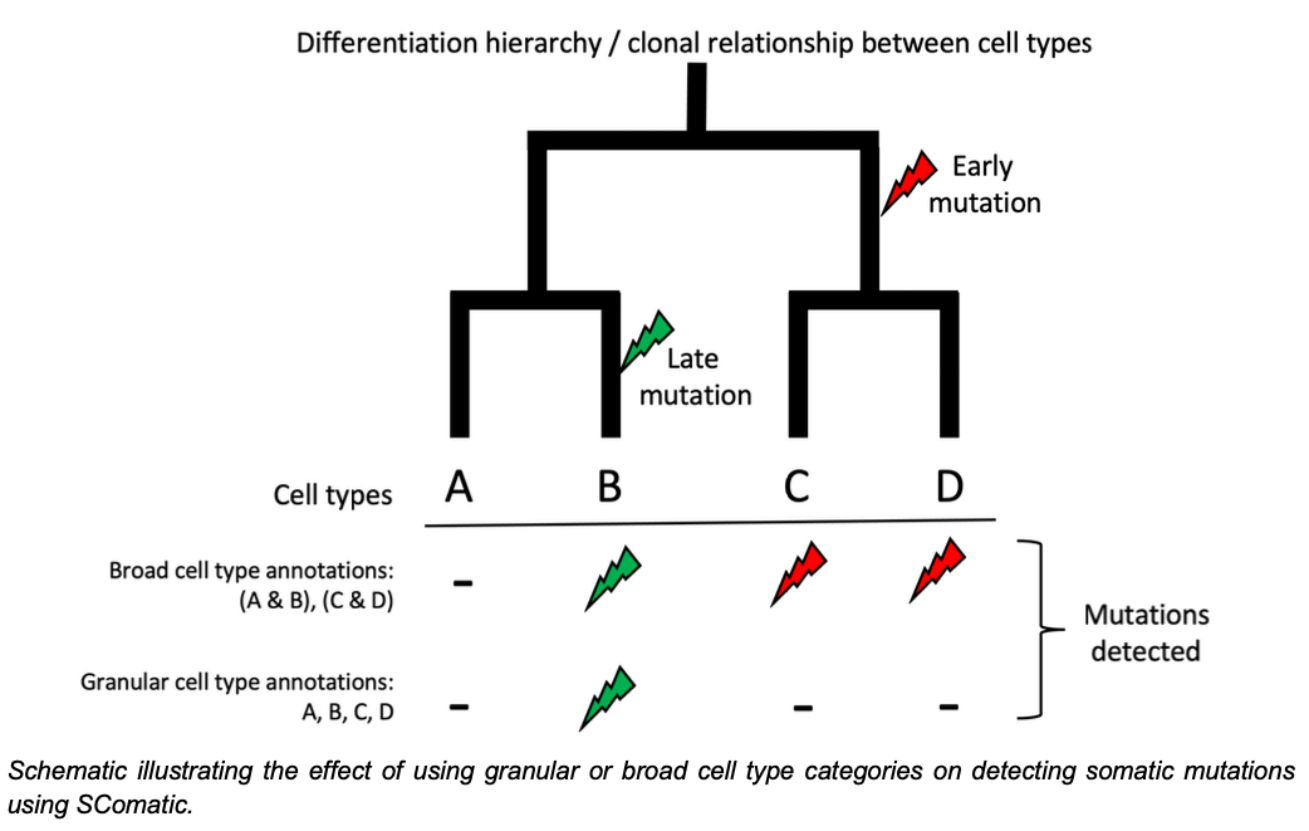

Supplement: Supplement 3 [file Supplemental_Code.zip › LongSom-paper_version/SComatic/docs/Granularity_plot.png]
